# Assessment of myocardial and LV blood pool post-contrast T1 evolution: comparison between healthy subjects and patients with hypertrophic cardiomyopathy

**DOI:** 10.1186/1532-429X-15-S1-E44

**Published:** 2013-01-30

**Authors:** Nadjia Kachenoura, Laila Besson-Hajji, Martin J Graves, Scott Reid, Golmehr Ashrafpoor, Laurent Macron, Arshid Azarine, Alban Redheuil, Elie Mousseaux

**Affiliations:** 1 INSERM U678, Paris, France; 2 Cardiovascular Radiology department, Europeen Hospital Georges Pompidou, Paris, France; 3 Cambridge University Hospitals NHS Foudation Trust, Cambridge, UK; 4 GE Healthcare, Cambridge, UK

## Background

The choice of post contrast acquisition time is crucial to optimize T1 mapping. Indeed, equilibrium is required for a reliable characterization of interstitial myocardial fibrosis. The majority of studies regarding T1 equilibrium were performed on healthy subjects. Accordingly, the aims of our study were 1) to assess post-contrast T1 Kinetics in HCM patients, in comparison to healthy volunteers, and 2) to determine acquisition times that enable a better differentiation between the two groups.

## Methods

We studies 14 HCM patients (10 males, age:58±12 years, myocardial wall thickness=12±4 mm, Heart rate=61±9 bpm), diagnosed by echocardiography, and 9 healthy volunteers (4 males, age:33±16 years, myocardial wall thickness=5±1 mm, Heart rate=71±10 bpm). Patients with arrhythmia or renal failure were excluded. Modified Look-Locker Inversion Recovery (MOLLI) sequences were acquired on a mid-ventricular short axis slice before contrast and every 5 minutes after a bolus injection (0.2 mmol/Kg, Dotarem) over 20 minutes. Segmental T1 values were calculated after myocardial delineation on each T1 map. Furthermore, presence of LGE was assessed visually in 20 segments from 2D LGE images.

## Results

**Figure 1:**
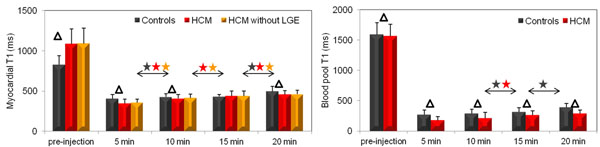
Left. Mean T1 values and their standard deviations calculated over all myocardial segments of controls (54 segments). HCM patients (84 segments) and HCM patients without LGE segments (64 segments) before and after injection. Right. T1 values and their standard deviations calculated within the LV cavity of controls and HCM patients before and after injection. Star indicated statistical significance according to a paired Wilcoxon test. Δ indicated statistical significance according to Mann Whitney test.

Pre-contrast myocardial T1 values were significantly (p<0.0001) higher in the HCM group (Figure 1). Myocardial T1 values decreased drastically in HCM patients at 5 minutes, and were significantly lower than myocardial T1 values in controls (p<0.0001). In controls, myocardial T1 reached steady state between 10 and 15 minutes while T1 values increased progressively and significantly in HCM. Of note, at 15 minutes, which is the acquisition time used in several studies, no significant differences in myocardial T1 were found between the HCM and control groups. At 20 minutes, myocardial T1 was significantly higher in controls reflecting a normal myocardial contrast wash-out (p=0.003). Similar results were found after excluding segments with LGE in HCM patients. Similar trends in the LV cavity T1 changes were observed between the 2 groups.

## Conclusions

While differences in myocardial T1 values were found to be highly significant between HCM and controls pre contrast and at 5 and 20 minutes after injection, no significant differences were found at 15 minutes. Accordingly, tailored acquisition times may be necessary for the characterization of myocardial interstitial fibrosis in differing conditions.

## Funding

None.

